# Supplementary material for: Acceptance and compliance with micronutrient powder (MNP) among children aged 6–23 months in northern Nigeria
Source: PLOS Glob Public Health. 2022 Oct 17;2(10):e0000961. doi: 10.1371/journal.pgph.0000961 (PMC10022258; doi:10.1371/journal.pgph.0000961)
Supplement: S1 Dataset — (ZIP) [file pgph.0000961.s008.zip › S8_Dataset/Spot Checks.zip!/Spot Checks/040715_A_SO_BA_2_SC.pdf]

SPOT CHECK RECORDING FORM

State: _ADAMAWA_ LGA: _SONG________________ Community: __GALANGWA____

Sex of index child: ___FEMALE___ Age of index child (in months): _13 MONTHS___

Spot Check Information

1. **Date of MNP distribution to household** (circle option below based on LGA name above)**:**
   - **Kebbi:** BirninKebbi&Jega LGA: **June 16**
   - **Kebbi:** Danko-wasagu: **June 17**
   - **Adamawa:**Song, Ganye: **June 18**
   - **Adamawa:**Guyuk **June 19**
2. **Date of Spot Check Observation: __04th July, 2015_________________**
3. **Number of sachets remaining**(Ask the caregiver to show you and count the specific number of sachets remaining)**:**

   42

4. **Number of sachets that <u>should remain</u> using one MNP sachet per day** (Analyst only)**:**

   45

5. **Number of sachets remaining / number of sachets should be remaining**(Analyst only)**:**

____42_____ / ____45_____ = ____93.3_____ %

Probes

Question 1. You have **XX** MNP sachets remaining. Explain how easy or difficult it was to use the MNP product on a daily basis for your child 6 – 23 months? (Circle one answer)

**Very Difficult**	**Difficult**	**Easy**	**Very Easy**

Question 2. Describe any specific challenges that you faced that made the MNP difficult to use on a daily basis?

Question 3. Describe specific aspects of the MNP product that you or your child enjoyed that made it easier to use on a daily basis?

The powder is to be used with kunu which is easy and fast to prepare. It also goes with food taken as breakfast were one is still strong to prepare the food. The MNP also dissolves well with the food and doesn’t affect the taste. So the child barely notice any changes in her food

Question 4. What questions do you have about the MNP based on your experience using it?

The child had been experiencing diarrhea since we started using the MNP, though it’s subsiding gradually. It there any reason to panic or use any other drug to treat the diarrhea?

Question 5. What specific recommendations do you have that we can do as a program to make the MNP easier to use on a daily basis?

Presently, I have no idea because you’ve tried so far. My concern is continuity of the program so that the whole community will benefit from it.
